# MTA2 as a Potential Biomarker and Its Involvement in Metastatic Progression of Human Renal Cancer by miR-133b Targeting MMP-9

**DOI:** 10.3390/cancers11121851

**Published:** 2019-11-23

**Authors:** Yong-Syuan Chen, Tung-Wei Hung, Shih-Chi Su, Chia-Liang Lin, Shun-Fa Yang, Chu-Che Lee, Chang-Fang Yeh, Yi-Hsien Hsieh, Jen-Pi Tsai

**Affiliations:** 1 Institute of Biochemistry, Microbiology and Immunology, Chung Shan Medical University, Taichung 40201, Taiwan; kevin810647@gmail.com (Y.-S.C.); hiking003@hotmail.com (C.-L.L.); yehchangfang@yahoo.com.tw (C.-F.Y.); 2 Division of Nephrology, Department of Medicine, Chung Shan Medical University Hospital, Taichung 40201, Taiwan; a6152000@ms34.hinet.net; 3 School of Medicine, Chung Shan Medical University, Taichung 40201, Taiwan; 4 Whole-Genome Research Core Laboratory of Human Diseases, Chang Gung Memorial Hospital, Keelung 20401, Taiwan; ssu1@cgmh.org.tw; 5 Department of Dermatology, Drug Hypersensitivity Clinical and Research Center, Chang Gung Memorial Hospital, Linkou 24451, Taiwan; 6 Institute of Medicine, Chung Shan Medical University, Taichung 40201, Taiwan; ysf@csmu.edu.tw; 7 Department of Medicine Research, Buddhist Dalin Tzu Chi Hospital, Chiayi 62247, Taiwan; dm731849@tzuchi.com.tw; 8 Department of Biochemistry, School of Medicine, Chung Shan Medical University, Taichung 40201, Taiwan; 9 Clinical laboratory, Chung Shan Medical University Hospital, Taichung 40201, Taiwan; 10 School of Medicine, Tzu Chi University, Hualien 97010, Taiwan; 11 Division of Nephrology, Department of Internal Medicine, Dalin Tzu Chi Hospital, Buddhist Tzu Chi Medical Foundation, Chiayi 62247, Taiwan

**Keywords:** renal cell carcinoma, metastasis, MTA2, MMP-9, miR-133b

## Abstract

Metastasis-associated protein 2 (MTA2) was previously known as a requirement to maintain malignant potentials in several human cancers. However, the role of MTA2 in the progression of renal cell carcinoma (RCC) has not yet been delineated. In this study, MTA2 expression was significantly increased in RCC tissues and cell lines. Increased MTA2 expression was significantly associated with tumour grade (*p* = 0.002) and was an independent prognostic factor for overall survival with a high RCC tumour grade. MTA2 knockdown inhibited the migration, invasion, and in vivo metastasis of RCC cells without effects on cell proliferation. Regarding molecular mechanisms, MTA2 knockdown reduced the activity, protein level, and mRNA expression of matrix metalloproteinase-9 (MMP-9) in RCC cells. Further analyses demonstrated that patients with lower miR-133b expression had poorer survival rates than those with higher expression from The Cancer Genome Atlas database. Moreover, miR-133b modulated the 3′untranslated region (UTR) of MMP-9 promoter activities and subsequently the migratory and invasive abilities of these dysregulated expressions of MTA2 in RCC cells. The inhibition of MTA2 could contribute to human RCC metastasis by regulating the expression of miR-133b targeting MMP-9 expression.

## 1. Introduction 

Renal cell carcinoma (RCC), which accounts for more than 90% of new cases of kidney cancer, is the most lethal genitourinary cancer, with limited median survival time and overall survival when advanced or distant metastasis occurs [1,2]. According to molecular medicine, genetics and clinical response help to determine the cell type of RCC [3,4], and clear cell RCC (ccRCC) are the most common subtypes and account for the highest RCC mortality and incidence rates [5]. In recent years, tyrosine kinase inhibitors (TKI) towards the vascular endothelial growth factor (VEGF), the mammalian target of rapamycin (mTOR) inhibitor, clustered, regularly interspaced short palindromic repeats-Cas9 (CRISPR-Cas9), small molecule inhibitors, and immune checkpoint inhibitors have had promising clinical outcomes against advanced RCC [6,7,8,9]. Reports have shown that using adjuvant sunitinib in high-risk RCC patients after nephrectomy resulted in median disease-free survival [10]. Therefore, the development of potential and novel molecular targets for treating advanced or metastatic RCC has currently become a critical topic. Metastasis-associated protein 2 (MTA2) is a central component of the Mi-2/nucleosome remodeling and deacetylase (NuRD) complex and precisely controls cytoskeleton reorganisation at the transcriptional level; moreover, it is closely associated with tumour progression and metastasis [11]. MTA2 overexpression has been observed in several human cancers and is associated with tumour invasion capacity, metastasis, and poor prognosis [12]. In gastric cancer, MTA2 can be transcriptionally regulated by specificity protein 1 (Sp1), and MTA2 expression is closely related to tumour invasion, lymph node metastasis, and Tumor-Node-Metastasis (TNM) staging [13]. MTA2 is expressed in aggressive lung cancer, and its increased expression is correlated with poor prognosis [14]. In estrogen receptor-alpha–negative breast cancer, MTA2 expression is associated with poor prognosis and enhanced metastasis in vitro and in vivo through Rho pathway activation [15].

A family of zinc-dependent endopeptidases with matrix metalloproteinases (MMPs) are the most critical to targeting various extracellular matrix (ECM) or basement membrane components [16]. Malignant cells can destroy the intercellular connection, lyse the ECM, breach the basement membrane, invade the vasculature, and exhibit distant metastasis because of dysregulated MMP activity [17]. For example, studies on RCC have demonstrated that increased expression of matrix metalloproteinase-9 (MMP-9) or phosphorylated extracellular signal-regulated kinase (ERK) correspond to RCC severity, which is correlated with tumour size, TMN stage, invasion, distant metastasis, and cancer-specific survival [18,19]. Combination therapy and drug synergism for targeted glioma improved the antitumor activity of individual treatment approaches [20,21]. Dr. Tabouret et al., found that MMP2 and MMP9 can be used as biomarkers in predicting bevacizumab activity in high-grade glioma patients [22].

MicroRNAs (miRNA) are short noncoding RNAs containing 19–23 nucleotides. By binding partial sequence homology to the three prime untranslated region (3′-UTR) of target mRNAs, miRNAs can regulate gene expression at the posttranscriptional level and cause translational inhibition and mRNA degradation [23]. miR-133b belongs to a miRNA family which includes other miRNAs, such as miR-133a. Studies have indicated that miR-133b can regulate oncogenic transcripts of gastric, esophageal, or breast cancer by targeting Fascin Actin-Bundling Protein 1(FSCIN1)/Sp1 [24,25], the epidermal growth factor receptor (EGFR) [26] and SRY-Box Transcription Factor 9 (SOX9) [27], respectively. Moreover, aberrant miR-133b expression plays a role in RCC development by suppressing cell proliferation and migratory and invasive abilities by modulating MMP-9 expression [28], but the molecular mechanism of MTA2 regulating miR-133b involvement in RCC metastasis has not yet been elucidated. To the best of our knowledge, the potential modulation the progression roles of MTA2 and miR-133b in RCC remain unclear. This study investigates the clinicopathological implications of MTA2 and miR-133b by analysing RCC tissue data and examining the pathophysiological functions and underlying mechanisms of MTA2 regulation of miR-133b on human RCC cell progression.

## 2. Results

### 2.1. Expression and Effects of MTA2 in Human RCC and RCC Cells

**Figure 1:**
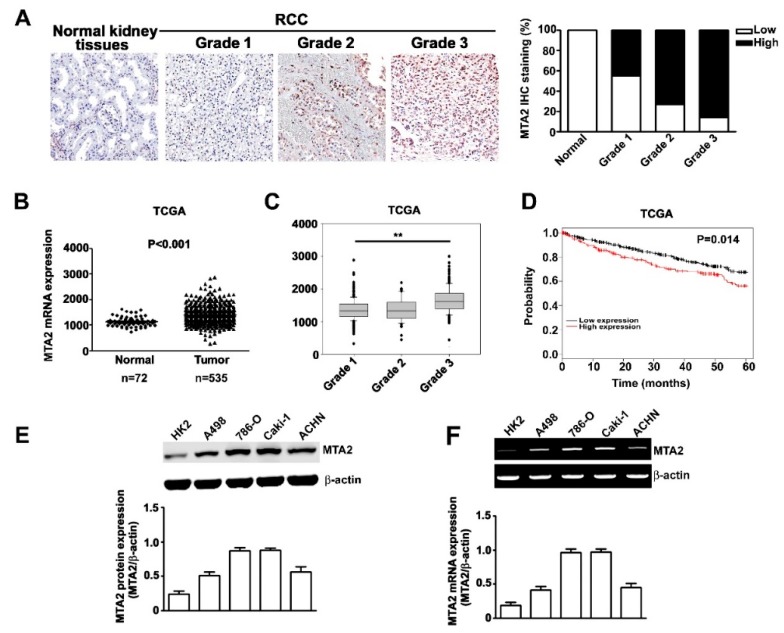
Expression and effects of metastasis-associated protein 2 (MTA2) in human renal cell carcinoma (RCC) and RCC cells. (**A**) Intensity of MTA2 expression in RCC grade 1, 2, and 3 and normal kidney tissues by using immunohistochemistry staining (×40). (**B**) MTA2 mRNA expression of RCC and normal tissue from The Cancer Genome Atlas (TCGA) datasets. (**C**) MTA2 mRNA expression in patients with RCC grade 1, 2, and 3. (**D**) Kaplan–Meier curve for overall survival of patients, categorised by low and high MTA2 expression. (**E**) Total lysates from HK2, A498, 786-O, Caki-1, and ACHN cells were isolated and analysed using western blotting to detect the individual expression of MTA2; β-actin was used as an internal control. (**F**) A reverse transcription polymerase chain reaction assay was applied to detect MTA2 mRNA expression. β-actin was used as an internal control for mRNA equal loading. Values are expressed as the mean ± SE of three independent experiments. ** *p* < 0.01 compared with normal kidney tissues.

MTA2 expression was examined through immunohistochemical (IHC) staining using a human kidney clear cell carcinoma tissue array procedure. As the tumour grade became severe, MTA2 expression was enhanced (Figure 1A). After staining, the correlation between MTA2 expression and clinicopathological parameters was analysed using data from 99 patients with RCC (Table 1). Patients diagnosed with grade 2 or 3 cancer had a significantly higher percentage of MTA2 expression compared with those diagnosed with grade 1 (*p* = 0.002). However, no significant association was observed between MTA2 expression and other parameters, such as tumour stage, age, or gender (Table 1). By using The Cancer Genome Atlas (TCGA) database, we observed higher mRNA expression of MTA2 in tumour tissues than in normal tissues (Figure 1B) and in higher tumour grades than in lower grades (Figure 1C). We further examined whether MTA2 expression was correlated with the postoperative survival of patients with RCC by using Kaplan–Meier survival analyses. Patients with RCC who had high MTA2 expression had a significantly lower survival rate compared with those with low MTA2 expression (*p* = 0.014, Figure 1D). Therefore, MTA2 expression level can serve as an independent prognostic factor for patients with RCC. Furthermore, western blot analysis and reverse transcription polymerase chain reactions (RT-PCR) were conducted to detect MTA2 expression in four RCC cell lines (A498, 786-O, Caki-1, and ACHN) and normal renal tubular cells (HK2 cells). RCC cell lines had a relatively high protein and mRNA expression of MTA2 compared with HK2 cells, (Figure 1E,F) indicating that the overexpression of MTA2 is involved in RCC. 

### 2.2. Effect of MTA2 Knockdown on RCC Cell Proliferation

**Figure 2:**
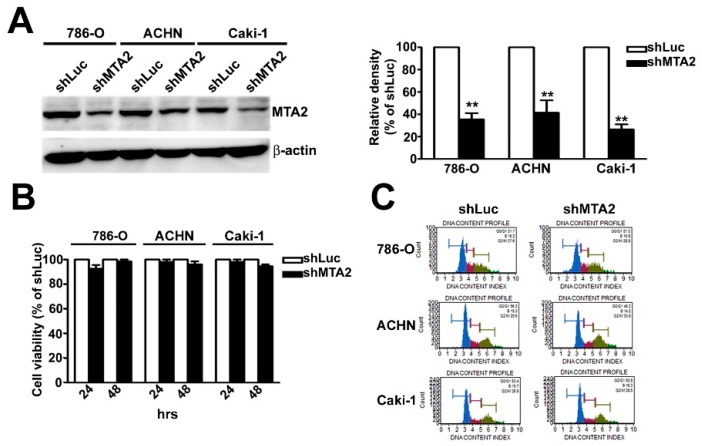
Metastasis-associated protein 2 (MTA2) knockdown did not influence viability or proliferation of renal cell carcinoma (RCC) cells. (**A**) MTA2 knockdown expression in shLuc or shMTA2 of 786-O, ACHN, and Caki-1 cells was verified using western blotting. (**B**) Cell viability of shLuc or shMTA2-786-O, ACHN, and Caki-1 cells was evaluated using an MTT assay after 24 and 48 h. (**C**) Flow cytometry analysis of shLuc–or shMTA2–786-O, ACHN, and Caki-1 cells. *β*-Actin was used as an internal control for protein equal loading. Values are expressed as the mean ± SE of three independent experiments. ** *p* < 0.01 compared with shLuc cells.

We examined the biological function of MTA2 in RCC cells by using a short hairpin RNA (shRNA) assay. MTA2 knockdown (shMTA2) inhibited MTA2 expression in three cell lines compared with short hairpin Luc (shLuc) cells in western blot analysis (Figure 2A). To further explore the influence of MTA2 on cell proliferation, we determined whether MTA2 knockdown had a cytotoxic effect on RCC cells using an (3-(4,5-Dimethylthiazol-2-yl)-2,5-diphenyltetrazolium bromide; MTT) MTT assay. We observed no difference in cell viability between shMTA2–RCC and shLuc–RCC cells (Figure 2B). Moreover, no differences in cell cycle distribution between shMTA2–RCC and shLuc–RCC cells were detected in the flow cytometry analysis (Figure 2C). Hence, MTA2 knockdown did not affect the proliferation of RCC cells.

### 2.3. Effect of MTA2 Knockdown on RCC Cell Metastasis in Vitro and in Vivo

**Figure 3:**
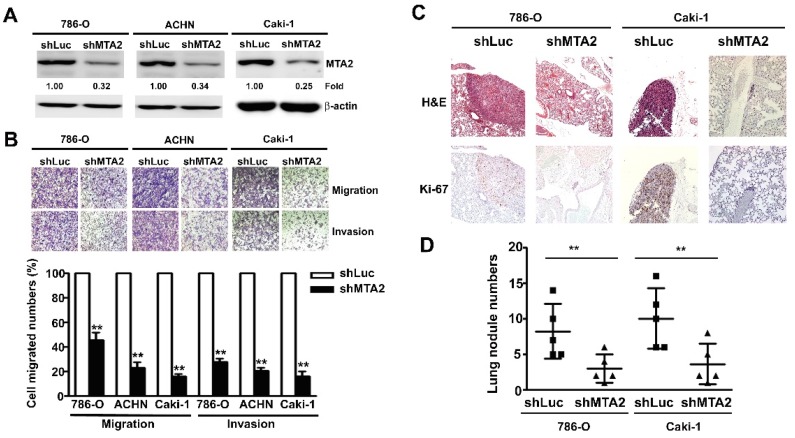
Metastasis-associated protein 2 (MTA2) knockdown inhibited migration and invasion of renal cell carcinoma cells and suppressed tumour metastasis in vivo. (**A**) MTA2 knockdown expression in 786-O, ACHN, and Caki-1 cells was verified using western blotting. (**B**) The migration and invasion abilities of shLuc and shMTA2-786-O, -ACHN, and –Caki-1 cells were determined using migration and Matrigel invasion assay. Cells in the lower surface of the Borden chamber were stained and photographed under a light microscope. The quantification of migrated cells are presented as a histogram. (**C**) Representative images of hematoxylin and eosin staining and Ki-67 expression in the shLuc and shMTA2 groups of 786-O and Caki-1 cells. (**D**) Considerably fewer metastatic lung colonies were observed in the shMTA2 group than in the shLuc group of 786-O and Caki-1 cells. Values are expressed as the mean ± standard error (SE) of three independent experiments. ** *p* < 0.01 compared with the shLuc cells.

After MTA2 knockdown, RCC cells (786-O, Caki-1, and ACHN) exhibited significantly reduced MTA2 expression using western blot analysis (Figure 3A). The quantification analysis demonstrated that migratory and invasive abilities were markedly reduced in shMTA2–RCC cells compared with shLuc–RCC cells (Figure 3B). To examine the effects of MTA2 on the distant metastasis abilities of RCC in vivo, we injected shLuc– and shMTA2–786-O or Caki-1 cells into the tail vein of mice. The growth of tumours stained with hematoxylin and eosin (H&E) and the expression of Ki-67 in the shMTA2 groups by using IHC assay were markedly lower than those observed in the shLuc groups (Figure 3C). Lung nodules were counted after sacrificing these mice, and markedly fewer nodules were observed in the shMTA2–786-O and shMTA2–Caki-1 cells than in shLuc–786-O and shLuc–Caki-1 cells (Figure 3D). Thus, MTA2 played a central role in regulating distant metastasis in RCC.

### 2.4. Effect of MTA2 Knockdown on MMP-9 Expression in RCC Cells

**Figure 4:**
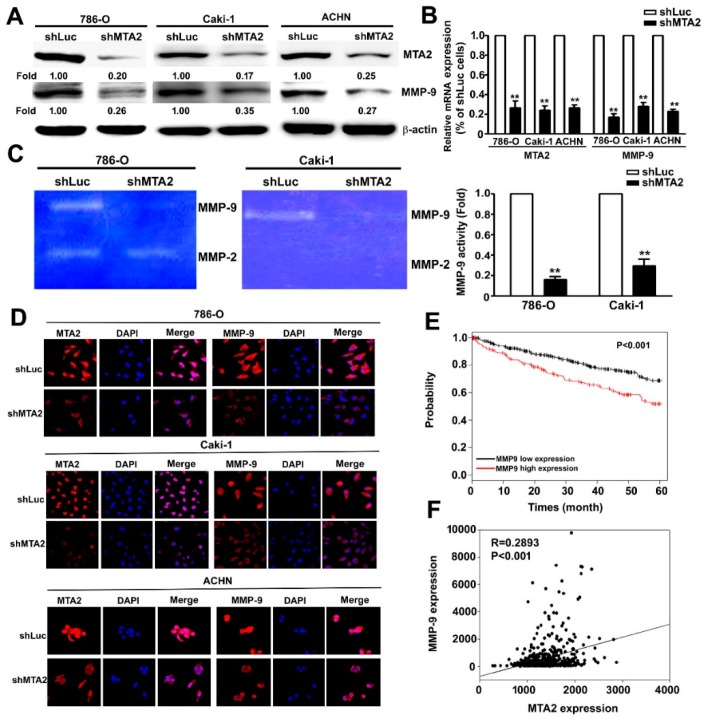
Metastasis-associated protein 2 (MTA2) knockdown inhibited the activity, expression, and mRNA levels of matrix metalloproteinase-9 (MMP-9) in renal cell carcinoma cells. (**A**, **B**) Total lysates and mRNA from shLuc– or shMTA2–786-O, Caki-1, and ACHN cells were isolated and analysed using western blotting and quantitative reverse transcription polymerase chain reaction assay to detect individual expression of MTA2 and MMP-9. *β*-Actin and GAPDH were used as internal controls. (**C**) Conditioned media were collected, and MMP-2 and MMP-9 activities were measured using gelatin zymography and quantified through densitometry. (**D**) shLuc- and shMTA2-786-O, -Caki-1, and -ACHN cells were stained with anti-MTA2 and anti-MMP-9 antibodies by using immunofluorescence staining, and cell nuclei (blue) were counterstained using 4’,6-diamidino-2-phenylindole (DAPI) reagent. (**E**) The Cancer Genome Atlas (TCGA) datasets was used to create the Kaplan–Meier curve portraying the overall survival of patients based on low or high MMP-9 expression. (**F**) Linear trend of the correlation between MTA2 and MMP-9 illustrated using the TCGA datasets. Values are expressed as the mean ± SE of three independent experiments. ** *p* < 0.01 compared with shLuc cells.

To identify the molecular mechanism of MTA2 in the invasive behaviour of RCC, western blot analysis, quantitative reverse transcription-polymerase chain reaction (qRT-PCR) assay, and gelatin zymography demonstrated that MTA2 knockdown significantly decreased the protein, mRNA, and activity expression of MMP-9 in 786-O, Caki-1, and ACHN cells, but was not involved in MMP-2 (Figure 4A–C). The immunofluorescence assay results were similar (Figure 4D). Regarding the function of MTA2, we found that overexpressed MTA2 in HK2 cells was increased the protein and mRNA of MTA2 and MMP-9, compared with Neo-HK2 cells (Supplemental Figure S1A, S1B). Furthermore, migratory and invasive capacity in MTA2-overexpressing HK2 cells was significantly higher than that in Neo-HK2 cells (Supplemental Figure S1C). In addition, Kaplan–Meier survival and log rank analyses suggested that patients with RCC and high MMP-9 expression had lower survival rates compared with those with low MMP-9 expression (*p* < 0.001, Figure 4E). MMP-9 expression was also positively correlated with MTA2 expression in patients with RCC (*p* < 0.001, Figure 4F). Therefore, MTA2 played a main role in migration and invasion of RCC cells by inhibiting MMP-9 expression.

### 2.5. MMP-9 as the Target Gene of miR-133b and Association with Poor RCC Prognosis.

**Figure 5:**
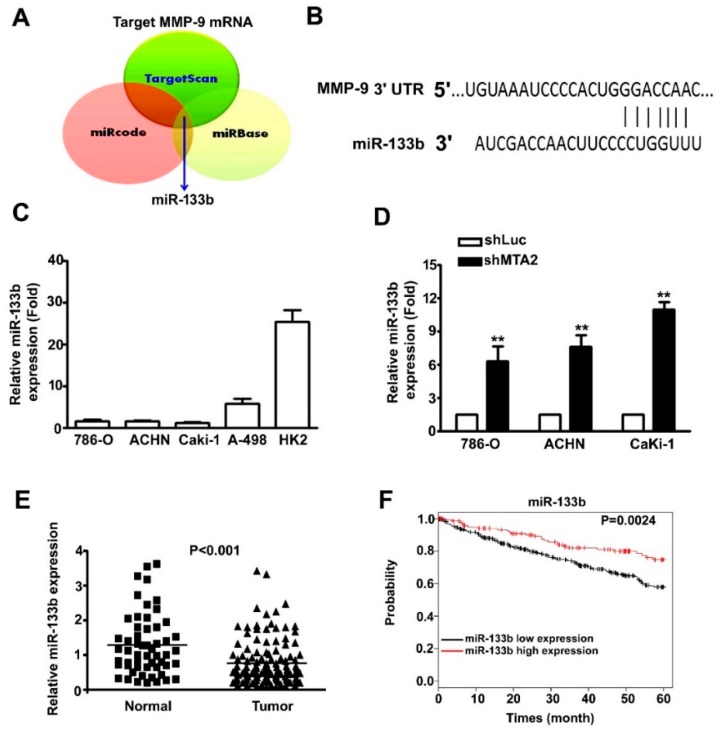
MMP-9 is the target for miR-133b and associated with poor renal cell carcinoma (RCC) prognosis. (**A**) Schematic of the proposed model depicting miR-133b targeting MMP-9 mRNA from three prediction datasets (TargetScan, miRcode, and miRBase). (**B**) Schematic of the predicted binding site of miR-133b at the 3′-UTR of MMP-9 promoter. (**C**) miR-133b expression in four RCC cells and normal kidney HK2 cells was measured using quantitative polymerase chain reaction (qPCR) assay. (**D**) miR-133b expression was detected in shLuc and shMTA2-RCC cells using qPCR. (**E**) miR-133b expression in tumour and normal tissues from The Cancer Genome Atlas RCC datasets. (**F**) Kaplan–Meier curve for the overall survival of patients with RCC categorised by low and high miR-133b expression. ** *p* < 0.01 compared with shLuc cells.

miR-133b target sites on the 3′-UTR regions of MMP-9 were identified using TargetScan, miRcode, and miRbase analytical programmes (Figure 5A). The MMP-9 3′-UTR promoter contained an miR-133b target sequence (Figure 5B). The quantitative polymerase chain reaction (qPCR) assay revealed a lower level of miR-133b in three RCC cells (786-O, Caki-1, A-498, and ACHN) than in HK2 cells (Figure 5C). In addition, miR-133b expression was increased in three shMTA2–RCC cells compared with in shLuc–RCC cells (Figure 5D). Using the TCGA database, we observed higher miR-133b expression in normal tissues compared with that in tumour tissues (Figure 5E). Kaplan–Meier survival analyses revealed that patients with RCC and low miR-133b expression had lower survival rates compared with those with high expression (*p* = 0.0024, Figure 5F). In summary, miR-133b could play a role in RCC development by regulating MMP-9 expression.

### 2.6. Effect of miR-133b on MMP-9 Expression Involved in Knockdown MTA2-Inhibiting RCC Cell Metastasis

**Figure 6:**
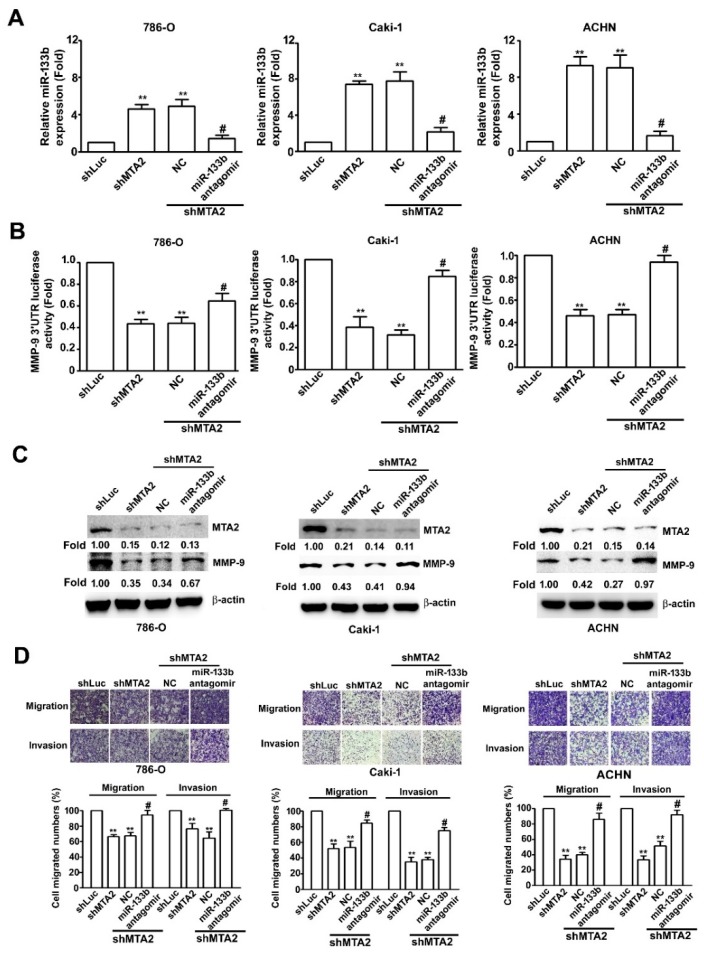
Effects of miR-133b on migratory and invasive ability in shMTA2-renal cell carcinoma (RCC) cells. (**A**) miR-133b expression in shMTA2–RCC cells after transfection with miR-133b antagomir or negative control (NC) were measured using RT-qPCR assays. (**B**) Luciferase activity assay. (**C**) Protein expressions of MTA2 and MMP-9 were detected using western blotting. *β*-Actin was used as an internal control. (**D**) Migration and invasion abilities of RCC cells were determined using migration and Matrigel invasion assays. Cells in the lower surface of the Borden chamber were stained and photographed under a light microscope at 400× magnification. Values are expressed as the mean ± SE of three independent experiments. ** *p* < 0.01 compared with the shLuc cells. # *p* <0.01 compared with shMTA2. NC, miR-133b negative control.

To clarify the regulatory effects of miR-133b on RCC metastasis progression, we attempted to validate whether miR-133b could regulate the effects of MTA2 on modulating MMP-9 expression and metastasis ability in RCC cells. RT-qPCR, luciferase reporter assay, and western blotting were performed. Increased miR-133b expression in shMTA2 and shMTA2-NC-RCC cells that were treated with miR-133b antagomir significantly inhibited miR-133b expression in shMTA2-RCC cells (Figure 6A). In addition, shMTA2–RCC cells expressed lower MMP-9 3′-UTR promoter activity than shLuc–RCC cells. Treatment with miR-133b antagomir significantly inhibited miR-133b expression and reversed MMP-9 3′-UTR promoter activity induced by shMTA2 compared with those in shMTA2-NC cells (Figure 6B). Similar results were achieved with western blotting (Figure 6C). Moreover, transfection with miR-133b antagomir significantly increased migratory and invasive abilities in shMTA2–RCC cells compared with those in shMTA2-NC cells (Figure 6D). To further confirm the tumour-suppressing role of miR-133b, we transfected NC- or antagomir-133b into HK2 cells, which exhibited relatively high miR-133b expression among RCC cells. A western blotting assay revealed a significantly higher MMP-9 expression in antagomir-133b–transfected HK2 cells than in NC-transfected cells (Supplementary Figure S2A) and inhibition of endogenous miR-133b expression by using RT-qPCR assay (Supplementary Figure S2B). The in vitro migration and invasion assays suggested an increase in the migration and invasion ability of antagomir-133b-transfected HK2 cells compared with that of NC-transfected cells (Supplementary Figure S2C). These studies indicated that MTA2 can affect RCC metastasis through miR-133b targeting of MMP-9 expression. 

## 3. Discussion

This study examined the hypothesis that the biological function and molecular mechanism of MTA2 induces miR-133b target MMP-9 expression in RCC metastasis progression. Our results indicated that (1) MTA2 expression was increased in RCC cells and was markedly correlated with high grade and poor survival rates of patients with RCC; (2) MTA2 did not affect RCC cell proliferation or cell cycle distribution; (3) MTA2 regulated the tumour metastasis of RCC cells and modulation of MMP-9 expression in vitro and in vivo; (4) miR-133b and MMP-9 expression in patients with RCC was negatively correlated with poor survival rates; (5) MTA2 knockdown inhibited RCC metastasis by targeting miR-133b and MMP-9 pathways. These results demonstrated the role of MTA2 in RCC metastasis, which is of tremendous help in creating new strategies against RCC metastasis at molecular translational levels.

MTA2 has been linked to tumour invasion depth, regional lymph node metastasis, distant metastasis, and poor long-term survival rates independent of age or gender in patients with esophageal squamous cell carcinoma [29], gastric cancer [13], non-small-cell lung cancer [30], and colorectal cancer [31]. Consistent with these studies, we observed higher MTA2 expression in tumour tissues compared with normal tissues and in all the RCC cell lines. Moreover, high MTA2 expression was markedly correlated with tumour grades and indicated low survival rates in accordance with the clinical pathologic data from our patients and TCGA database (Figure 1). Hence, we concluded that MTA2 overexpression has potential as an oncogenic factor for predicting the prognosis of patients with RCC.

Invasion and metastasis are characteristic features of cancer cells and a key impediment of effective prognosis [32]. In RCCs, overexpression of MMP-1, MMP-2, and MMP-9 is linked to tumour stage, histological grade, progression, invasion of microvasculature, and distant metastasis [33]. Targeting MTA2 with a short hairpin RNA could reduce cell proliferation and inhibit metastasis by downregulating MMP-2 or MMP-9 expression in breast cancer [34] and glioma cells [35]. Other reports have demonstrated that MTA2 overexpression could activate AKT and upregulate MMP-7 expression in nasopharyngeal carcinoma cells [36]. On the basis of these studies, we demonstrated that MTA2 knockdown could decrease the expression of MMP-9 and the invasive, migratory, and metastatic abilities of RCC cells. Therefore, MTA2 could influence the malignant factor that modulates MMP-9 expression for RCC.

Reports have demonstrated the downregulated expression of miR-133b, which is involved in the progression and in negative regulation of proliferation and metastasis in various tumours, such as glioma [37], breast cancer [27], prostate cancer [38], and bladder cancer [39]. In gastric cancer, miR-133b expression was negatively associated with lymph node metastasis, and miR-133b targeting Gli-1 markedly inhibited gastric cancer metastasis [40]. Kano et al. [24], revealed that miR-133b reduced proliferation and invasion of esophageal squamous cells by inhibiting FSCN1 expression. In ovarian cancer, the overexpression of miR-133b targeted EGFR and inhibited proliferation and invasion abilities by decreasing the phosphorylation of ERK1/2 and AKT pathways [41]. Overexpression of miR-133b induces RCC cell apoptosis by counteracting Janus kinase 2 (JAK2)/ Signal transducer and activator of transcription 3 (STAT3) pathway phosphorylation [42]. In addition to these studies, miR-133b inhibited RCC cell proliferation and metastasis by targeting MMP-9 [28]. However, the depth of the molecular mechanisms of MTA2 modulating miR-133b in RCC metastasis remains unclear. Consistent with the aforementioned results, MTA2 knockdown inhibited RCC metastasis by targeting MMP-9 expression. To explore the role of miR-133b in MTA2 regulating RCC metastasis, we used a miR-133b antagomir to restore MMP-9 expression in shMTA2 RCC cells. Similarly, inhibition of miR-133b in HK2 cells, which exhibit relatively high levels of miR-133b, significantly enhanced HK2 cell migration and invasion when treated with miR-133b antagomir. Therefore, miR-133b could modulate the effects of MTA2 on RCC cell invasion and migration abilities predominantly by targeting MMP-9 expression. At a clinical level, TCGA database analysis and Kaplan–Meier survival analyses revealed that patients with RCC with low miR-133b expression had lower survival rates compared with those with high expression, and miR-133b may be a prognostic marker of RCC. However, whether miR-133b is a predictor of clinical outcome in RCC cancer warrants further investigation.

In summary, our data suggested that MTA2 was overexpressed in RCC tissues and cells and positively correlated with tumour grade and MMP-9 expression in vitro and in vivo. Moreover, MTA2 knockdown inhibited RCC metastasis by regulating miR-133b targeting of MMP-9, and miR-133b was negatively correlated with RCC progression. Therefore, MTA2 regulation of miR-133b may be a novel diagnostic and therapeutic target for RCC treatment.

## 4. Materials and Methods

### 4.1. Materials and Reagents

MTT (tetrazolium dye, 3-(4,5-dimethylthiazol-2-yl)-2,5-diphenyltetrazolium bromide), Giemsa solution, and DAPI (4′-6-diamidino-2-phenylindole) were purchased from Sigma-Aldrich (St. Louis, MO). Dulbecco’s Modified Eagle Medium: Nutrient Mixture F-12 (DMEM/F-12) medium powder was purchased from Gibco-Invitrogen Corporation (Gibco, Carlsbad, CA, USA), whereas the RPMI-1640 and minimum essential media (MEM) powder were purchased from HyClone (Pittsburgh, PA, USA). Fetal bovine serum (FBS), penicillin/streptomycin, and 0.25% trypsin were purchased from HyClone (Pittsburgh, PA, USA). The antibodies for western blotting and immunofluorescence assay against MTA2 (sc-55566) and β-actin (sc-69879) were purchased from Santa Cruz Biotechnology (Santa Cruz, CA, USA). Antibodies against MTA2 (ab171073) and MMP-9 (ab137867) were purchased from Abcam (Cambridge, UK). Antibodies against goat antirabbit immunoglobulin (IgG, AP132P) and goat antimouse IgG (AP124P) were purchased from Merck Millipore (Merck Millipore, Burlington, MA, USA). The human MTA2 plasmid was synthesized from the GENEWIZ company (Takeley, UK).

### 4.2. Human Kidney Clear Cell Carcinoma Tissue Array

Kidney clear cell carcinoma tissue array contained human kidney cancer specimens and normal kidney tissue (BC07115; US Biomax Inc., Rockville, MD, USA). Clinicopathological characteristics, such as gender, age, tumour grade, and tumour stage were obtained from medical records. Kidney clear cell carcinoma tissue arrays were detected using immunohistochemical (IHC) staining for MTA2 according to previous reports [43].

### 4.3. Cell Culture and shRNA Assay

Human renal cancer cell lines (786-O, Caki-1, A-498, and ACHN) and normal HK2 cells were purchased from the Bioresources Collection and Research Centre of the Food Industry Research and Development Institute (Hsinchu, Taiwan). The 786-O cell lines were cultured in RPMI-1640. A498, Caki-1, and ACHN cell lines were cultured in MEM. HK2 cell lines were cultured in DMEM-F12 media. All cell lines were supplemented with a medium containing 10% FBS (Gibco, USA) and 1% penicillin/streptomycin at a humidified atmosphere containing 5% CO_2_ at 37 °C. For the shRNA assay, the shMTA2 (MTA2-shRNA-pLKO.1) and shLuc (Luc-shRNA-pLKO.1) plasmids were purchased from RNAi core of Academia Sinica (Taipei, Taiwan). The MTA2 target sequences were 5′-AGGGAGTGAGGAGTGAATTAA-3′, and the pLKO.1-Luc was a scrambled control. We used puromycin (2 μg/mL) to select stably transduced RCC cells as previously reported [44].

### 4.4. RNA Isolation, RT-PCR, and QRT-PCR

The total RNA was isolated from cells by using TRIzol (Invitrogen, Waltham, MA, USA). RNA samples (1 μg) were reverse transcribed to cDNA using GoScript Reverse Transcription Mix (Promega, Madison, WI, USA). RT-PCR was conducted using GoTaq Green Master Mix (Promega, USA). The PCR reaction conditions were 30 s at 95 °C, 30 cycles of 30 s at 95 °C for denaturation, 30 s at 52 °C for annealing, 90 s at 72 °C for extension, and 10 min at 72 °C for the final extension. mRNA levels were detected using the SYBR Green PCR Master Mix (Promega, USA) and analysed using Applied Biosystems Step One Plus Real-Time PCR System (Applied Biosystems, Waltham, MA, USA), as described by the manufacturer. The primers for the RT-PCR were MTA2 (forward: 5′-GTTCTGGCAATACGGCGAGT-3′, reverse: 5′-CTTCGGCTGAATGCACAAAGA-3′) and β-actin (forward: 5′-ACTGGAACGGTGAAGGTGAC-3′, reverse: 5′-AGAGAAGTGGGGTGGCTTTT-3′). The primers for the qRT-PCR were MMP-9 (forward: 5′-ACGACGTCTTCCAGTACCGA-3′, reverse: 5′-TCATAGGTCACGTAGCCCAC-3′), and GAPDH (forward: 5′-CATCATCCCTGCCTCTACTG-3′, reverse; 5′-GCCTGCTTCACCACCTTC-3′). All reactions for RT-PCR and qRT-PCR were run in triplicate and normalised to the internal control products of β-actin and GAPDH.

### 4.5. Cell Viability Assay

The shLuc and shMTA2 cells were seeded into 24-well plates at a density of 2 × 10^4^ cells/well and cultured for 24 and 48 h. MTT reagents were used to determine cell viability by following the manufacturer’s protocol. Three wells were measured for cell viability in each treatment group. The absorbance value at a wavelength of 570 nm was used as an indicator of cell viability.

### 4.6. Cell Cycle Analysis

Cell cycle distribution was analysed using Muse Cell Analyser (Millipore, Hayward, CA, USA). The shLuc and shMTA2 cells were washed three times with ice-cold phosphate-buffered saline and fixed with 70% ethanol at −20 °C overnight. Then, cells were cultured in 50  μg/mL propidium iodide and 1 mg/mL RNase for 30 min at room temperature. Finally, the treated cells were analysed. At least 50,000 cells were acquired for each sample. The experiments were performed in triplicate.

### 4.7. In Vitro Cell Migration and Invasion Assays

In vitro migration assays were performed using Boyben chamber inserts containing an 8.0 μm polycarbonate membrane (Corning, New York, NY, USA). Membranes were coated with 5% Matrigel matrix (BD Biosciences, Bedford, MA, USA) to determine the tumour cell invasion. The shLuc and shMTA2 cells (2 × 10^4^ cells/well) in 50 μL of serum-free media were added to the upper chamber, and 35 μL of medium/well with 10% FBS was added to the lower chamber. After 24 h of incubation at 37 °C, the cells remaining in the upper membrane were completely removed by gentle swabbing, whereas the migrated or invaded cells were attached to the lower part of the membrane insert. The lower surface of the membrane was fixed in 95% methanol for 10 min and stained with 0.5% crystal violet for 30 min. The cells were then counted under a microscope in five different fields. All experiments were performed in triplicate.

### 4.8. MiR-133b Antagomir and MTA2 Plasmid Transfection

The shLuc and shMTA2 cells were seeded into 6 cm dishes at a density of 2 × 10^5^ cells/well for 24 h and transfected with the miR-133b antagomir and MTA2 plasmid by using the TurboFect transfection reagent (Thermo Fisher Scientific, Waltham, MA USA) as previously reported [45].

### 4.9. Western Blot Analysis

Total protein was extracted from the shLuc and shMTA2 cells. Protein concentration was determined using the Bradford method (Bio-Rad, Hercules, CA, USA). Primary antibodies, namely MTA2, MMP-9, and β-actin, were incubated overnight at 4 °C, washed twice, and then incubated with secondary antirabbit and antimouse IgG for 60 min. Immunoreactive bands were detected with a chemiluminescence kit (Millipore, Billerica, MA, USA) using the ImageQuant LAS 4000 mini according to the manufacturer’s instructions.

### 4.10. Immunofluorescence Assay

After seeding 1 × 10^4^ shLuc and shMTA2 cells/well in a Nunc Lab-Tek chambered cover glass (Thermo, USA) for 24 h, cells were washed twice with phosphate-buffered saline (PBS), fixed with 4% paraformaldehyde for 10 min, washed twice with PBS, permeabilised with PBS containing 0.1% Triton X-100 for 10 min, and blocked with 2% bovine serum albumin for 2 h. Primary antibodies against MTA2 and MMP-9 were incubated in 2% bovine serum albumin at 4 °C overnight, and second antibodies were incubated in 2% bovine serum albumin at room temperature for 2 h. DAPI was used as the counterstaining medium for the cell nucleus. The results were visualised using a Zeiss LSM 510 confocal microscope.

### 4.11. Gelatine Zymography

MMP activity in a serum-free medium after culturing with shLuc and shMTA2 cells in 786-O and Caki-1 was detected using 8% SDS-polyacrylamide gel electrophoresis (PAGE) containing 0.1% gelatin. After electrophoresis, the gel was washed with Tris-buffered saline containing 2.5% Triton X-100 and then incubated in a reaction buffer overnight. Finally, the gel was stained with Coomassie Brilliant Blue R-250.

### 4.12. TCGA Database and miRNA Prediction of Bioinformatic Analysis

Clinical mRNA expression data, such as overall survival status of patients with RCC, were downloaded from TCGA datasets. To identify the miR-133b target gene, we used miRBase (http://www.mirbase.org), miRcode (http://www.mircode.org/), and TargetScan (http://www.targetscan.org/) to surmise the miRNA binding site in the 3′-UTR of MMP-9.

### 4.13. Luciferase Reporter Assay

The shLuc and shMTA2 cells in 786-O, Caki-1, and ACHN cells were cotransfected with pGL4.13-MMP-9-3′UTR-wt/mut and pRL Renilla luciferase control reporter vectors by using the TurboFect transfection reagent. The 3′-UTR sequences of MMP-9 containing the miR-133b binding site were constructed in the pGL4.13 vector by using PCR assay. Luciferase activity was detected using the Dual-Luciferase Reporter Assay System (Promega, Madison, WI, USA). All experiment steps followed the protocol. The activity of pRL Renilla luciferase control reporter vectors was used as the internal control.

### 4.14. In Vivo Animal Model and Immunohistochemistry Analysis

Five-week-old C. B17 mice weighing approximately 20 g were obtained from the National Laboratory Animal Centre (Taipei, Taiwan). All animal experiments were conducted following the protocols approved by the Institutional Animal Care and Use Committee of Chung Shan Medical University (IACUC: 2120). The shLuc and shMTA2 cells (six mice/group) were injected into the tail veins at a density of 1 × 106 in 0.1 mL of saline. All mice were euthanised after 2 months, and lung tissues were resected. To investigate the metastasis ability of shLuc and shMTA2 cells, lung tissues were harvested, embedded with paraffin, fixed in formalin, and processed for IHC staining. The amount and size of shLuc and shMTA2 cells metastasised in the lungs were analysed using H&E and Ki-67 staining.

### 4.15. Statistical Analyses

Results are expressed as mean ± standard deviation. Differences between the two groups were analysed using Student’s t-test or one-way analysis of variance followed by the Tukey post hoc test. The correlation between MTA2 and MMP-9 expression was measured using Spearman’s correlation analysis. All statistical calculations were performed using SPSS 12.0. A *p*-value less than 0.05 or 0.01 was regarded as statistically significant.

## 5. Conclusions

This study is the first to demonstrate that MTA2 could serve as an indicator for predicting the prognosis of patients with RCC. Moreover, we highlighted that MTA2 could regulate the RCC process by modulating miR-133b targeting MMP-9 expression. Finally, we hypothesised that treatments for MTA2 or modulating the expression of miR-133b targeting MMP-9 are promising therapies in addition to current therapies for RCC.

## Figures and Tables

**Table 1:** Correlation between metastasis-associated protein 2 (MTA2) expression and clinicopathological characteristics of renal cancer patients.

Characteristic	Number of Patients (%)		*p* Value
	MTA2 Staining		
	Negative	Positive	
Total Number of Patients	40 (40.4)	59 (59.5)	−
Age (Year)			
<59	20 (31.3)	26 (68.7)	0.580
≥59	20 (32.1)	33 (67.9)	
Gender			
Male	13 (43.3)	17 (56.7)	0.347
Female	27 (39.1)	42 (60.9)	
Tumor Grade			
1	29 (54.7)	24 (45.3)	0.002
2 + 3	11 (23.9)	35 (76.1)	
Tumor Stage			
Ι	27 (36.5)	47 (63.5)	0.129
II + III	2 (10)	18 (90)	

## Supplementary Materials

The following are available online at https://www.mdpi.com/2072-6694/11/12/1851/s1, Figure S1: MTA2 promote the migration and invasion of HK2 cells through modulating MMP-9 expression; Figure S2: MiR-133b influences cell migration/invasion and MMP-9 expression in HK2 cells.
