# Supplementary material for: MTA2 as a Potential Biomarker and Its Involvement in Metastatic Progression of Human Renal Cancer by miR-133b Targeting MMP-9
Source: Cancers (Basel). 2019 Nov 23;11(12):1851. doi: 10.3390/cancers11121851 (PMC6966675; doi:10.3390/cancers11121851)
Supplement: Supplementary file 1 [file cancers-11-01851-s001.zip › cancers-639216-SI.docx]

**MTA2 as a Potential Biomarker and its Involvement in Metastatic Progression of Human Renal Cancer by miR-133b Targeting MMP-9**

**Supplementary Data**


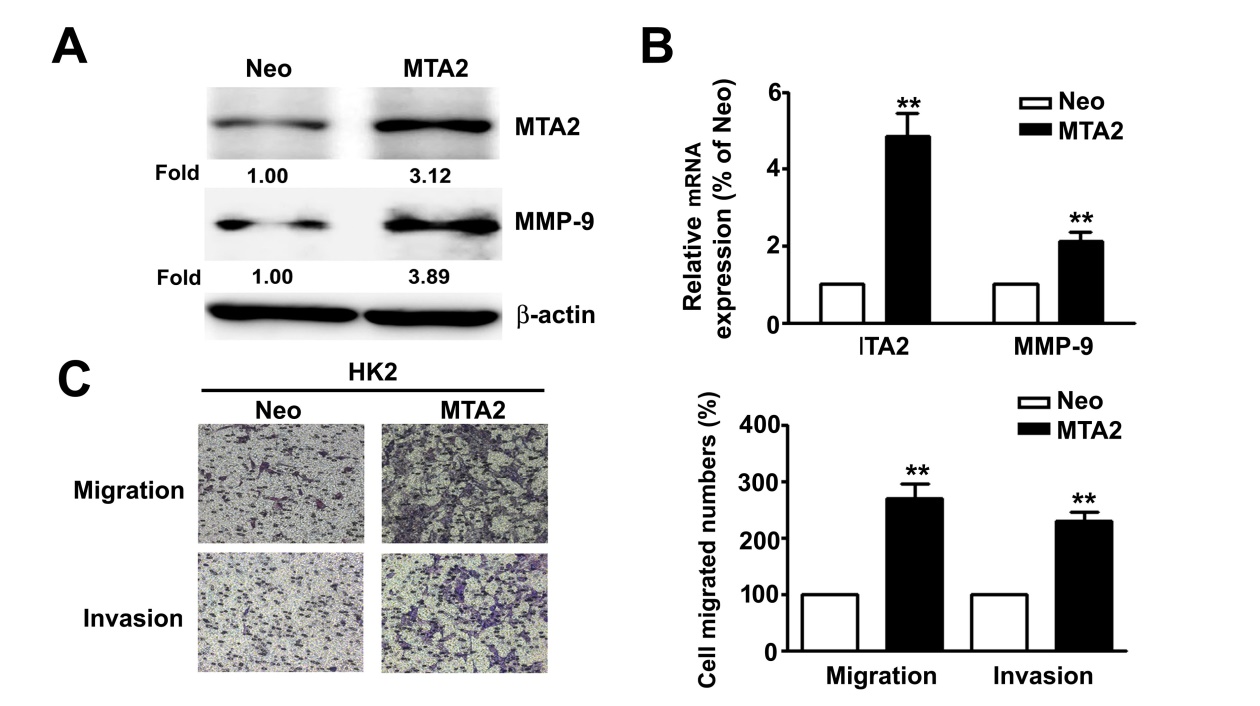


**Supplementary Figure S1.** **MTA2 promote the migration and invasion of HK2 cells through modulating MMP-9 expression.** **(A, B)** The protein expression of MTA2 and MMP-9 in MTA2 or Neo overexpressing-HK2 cells were detected by western blotting and RT-qPCR assay. **(C)** Cell migration and invasion were determined by migration and invasion assay. Values were expressed as the mean ± SE of three independent experiments. ** *P*<0.01 compared with the Neo cells.


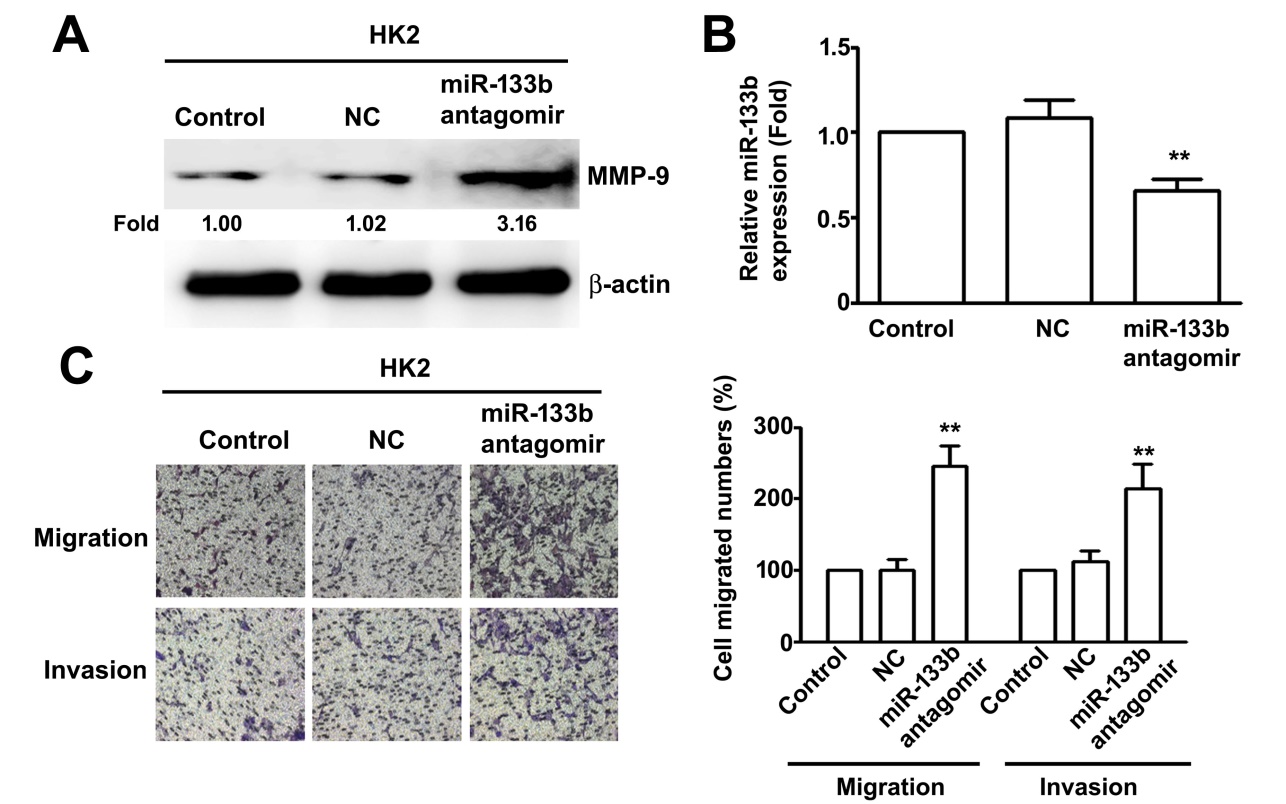


**Supplementary Figure S2. MiR-133b influences cell migration/invasion and MMP-9 expression in HK2 cells.** **(A)** The protein expression of MMP-9 in control, NC-transfected and miR-133b antagomir-transfected HK2 cells. **(B)** The miR-133b expression was detected by qPCR assay. **(C)** Cell migration and invasion were determined by migration and invasion assay. Values were expressed as the mean ± SE of three independent experiments. ** *P*<0.01 compared with the control cells.
